# Achieving Selective and Efficient Electrocatalytic Activity for CO_2_ Reduction on N-Doped Graphene

**DOI:** 10.3389/fchem.2021.734460

**Published:** 2021-08-19

**Authors:** Xiaoxu Sun

**Affiliations:** Jiangsu Key Laboratory of New Power Batteries, School of Chemistry and Materials Science, Nanjing Normal University, Nanjing, China

**Keywords:** density functional theory, N-doped graphene, CO_2_ reduction reaction, catalytic activity, Gibbs free energy

## Abstract

The CO_2_ electrochemical reduction reaction (CO_2_RR) has been a promising conversion method for CO_2_ utilization. Currently, the lack of electrocatalysts with favorable stability and high efficiency hindered the development of CO_2_RR. Nitrogen-doped graphene nanocarbons have great promise in replacing metal catalysts for catalyzing CO_2_RR. By using the density functional theory (DFT) method, the catalytic mechanism and activity of CO_2_RR on 11 types of nitrogen-doped graphene have been explored. The free energy analysis reveals that the zigzag pyridinic N- and zigzag graphitic N-doped graphene possess outstanding catalytic activity and selectivity for HCOOH production with an energy barrier of 0.38 and 0.39 eV, respectively. CO is a competitive product since its free energy lies only about 0.20 eV above HCOOH. The minor product is CH_3_OH and CH_4_ for the zigzag pyridinic N-doped graphene and HCHO for zigzag graphitic N-doped graphene, respectively. However, for Z-pyN, CO_2_RR is passivated by too strong HER. Meanwhile, by modifying the pH value of the electrolyte, Z-GN could be selected as a promising nonmetal electrocatalyst for CO_2_RR in generating HCOOH.

## Introduction

As one of the greenhouse gases, the continual accumulation of CO_2_ causes global warming, which significantly hinders the sustainable development of human society (Thomas et al., 2004; Lewis et al., 2006; Cook et al., 2010). The unbalanced CO_2_ emission and consumption is becoming a pressing issue (Kondratenko et al., 2013; Appel et al., 2013). In this aspect, CO_2_ electrochemical reduction reaction (CO_2_RR) by using the renewable energy sources (Yi et al., 2019; Wang et al., 2020; Lu et al., 2021) offers a promising way to produce fuels and value-added chemicals. Up to now, the major obstacle for CO_2_RR is the lack of electrocatalysts with high stability and efficiency. Particularly, the cathode electrocatalyst materials play a key role in the complicated product distribution of CO_2_RR (Lim et al., 2014; Zhu et al., 2016). Therefore, searching for suitable electrocatalysts for CO_2_RR is one of the hot topics in recent years. Till now, a lot of electrocatalysts for CO_2_RR have been studied, including noble metals (Zhu et al., 2013; Kang et al., 2014; Gao et al., 2015; Kim et al., 2015), base metals (Hori et al., 1985; Hori et al., 1986; Nie et al., 2013; Zhang et al., 2014a), alloys (Kim et al., 2014; Bai et al., 2017), and metal oxides (Lee et al., 2015; Ren et al., 2015). It is well known that Ag and Au are prone to produce CO *via* the two-electron reaction pathway (Zhu et al., 2013; Kim et al., 2015). In addition, Cu is recognized as a state-of-the-art CO_2_RR catalyst for generating multi-electron products, such as CO, HCOOH, CH_3_OH, and CH_4_ (Hori et al., 1985; Hori et al., 1986; Nie et al., 2013). However, the high cost, low efficiency due to the competitive hydrogen evolution reaction (HER), and high overpotential restrict their practical implementation and industrial-scale development in CO_2_RR (Lim et al., 2014).

To solve the above issues, metal-free electrocatalysts based on carbon materials have been studied, owing to their low cost, high stability, outstanding mechanical flexibility, and superior structural durability. The introduction of heteroatoms (such as N, B, and S) could not only modify the electronic structure of carbon materials but also contribute to take advantage of the existing defects appropriately (Wang X. et al., 2014). For N-doped carbon nanofibers (NCNFs), it shows negligible overpotential (0.17 V) and 13 times higher current density than bulk Ag catalyst for CO_2_RR (Kumar et al., 2013). In addition, N-doped carbon nanotubes (NCNTs) (Sharma et al., 2015), N-doped nanoporous carbon–carbon nanotube composite membrane (HNCM/CNT) (Wang et al., 2017), and polyethylenimine functionalized NCNTs have been proven to be highly active and stable electrocatalysts for CO_2_RR (Zhang et al., 2014b). Remarkably, N-doped graphene possesses excellent durability in the CO_2_RR process, achieving a maximum faradaic efficiency (FE) of 73% for formate with overpotential of 0.84 V (Wang et al., 2016). N-doped graphene quantum dots (NGQDs) could catalyze carbon dioxide into multicarbon hydrocarbons and oxygenates at high FE (up to 90%), with excellent selectivity (45% for ethylene and ethanol conversions) (Wu et al., 2016).

With respect to the active sites of nitrogen-doped carbon materials for CO_2_RR, it is a controversial issue among the pyridinic N, pyrrolic N, graphitic N, and the C adjacent to N. Generally, these potential active sites coexist in the carbon materials, which adds to the difficulty in identifying the active site. A theoretical study indicates that for CO_2_ electroreduction to CO on NCNTs, the optimal active site is pyridinic N, followed by pyrrolic N and graphitic N (Wu et al., 2015). Another study about CO_2_RR on NCNTs emphasizes that the presence of graphitic and pyridinic N defects remarkably increases the selectivity toward CO formation and decreases the absolute overpotential (Sharma et al., 2015). For N-doped graphene-like material/carbon paper electrodes (NGM/CP), the FE is as high as 93.5% in producing CH_4_, which is ascribed to the reactive pyridinic and pyrrolic N sites (Sun et al., 2016). A theoretical study suggested that COOH production on pyrrolic N3 is downhill by −0.21 eV, while it is uphill for pyridinic and graphitic N (Liu et al., 2016). Overall, both the experimental and theoretical studies indicate that N-doped carbon materials show significant catalytic performance of CO_2_RR.

Inspired by these studies, we studied CO_2_RR on N-doped graphene from the perspective of theoretical calculation in this work. To make a systematic comparison, N was doped into graphene at in-plane, zigzag edge, armchair edge, and pyrrolic edge sites, respectively. It would contribute to identifying the most dominant structure and providing a valuable design strategy for further activity enhancement in the experiment. In this study, the first-principle calculation has been performed to uncover the CO_2_RR reaction pathways and electrocatalytic activity on different edges of N-doped (zigzag edge, armchair edge, and pyrrolic edge) graphene structures within a unified thermodynamic reaction scheme.

## Computational Methods and Models

### Methods

The geometry optimization and energy calculations were performed within the density functional theory (DFT) framework (Kohn and Sham, 1965) by using the Vienna *ab initio* simulation package (VASP) (Kresse and Furthmüller, 1996a). The ion–electron interaction was described by the projector-augmented wave (PAW) potentials (Blöchl, 1994). The generalized gradient approximation parameterized by Perdew, Burke, and Ernzerhof was utilized as the exchange-correlation functional (Perdew et al., 1996). The kinetic energy cutoff of 400 eV was adopted for the plane-wave expansion. The armchair-edged ribbon, zigzag-edged ribbon, and periodic graphene slab were sampled with 4 × 1 × 1, 1 × 4×1, and 4 × 4 × 1 Monkhorst−Pack k-point grids (Delley, 2000), respectively. During the geometry optimization, all atoms were relaxed until the total energy was converged to 1.0 × 10^–5^ eV/atom, and the force was converged to 0.01 eV/Å. In addition, we considered the van der Waals (vdW) interactions by employing the semiempirical DFT-D2 forcefield approach (Grimme, 2006).

### Models

The lattice parameters of 8.52 Å × 24.6 Å and 25.6 Å × 9.84 Å were set to model the armchair-edged graphene nanoribbon (including pyrrolic edge) and zigzag-edged graphene nanoribbon, respectively. The lattice parameters of 9.84 × 9.84Å were adopted to model the periodic graphene slab. Perpendicular to all graphene structures, a vacuum layer of 15 Å was set, which was sufficiently large to minimize the image interactions.

The adsorption energy$\left(\Delta E_{\mathrm{ads}}\right)$of adsorbates was defined as follows:

$$\Delta E_{\mathrm{ads}}=E_{\mathrm{substrate+adsorbate}}-\left(E_{\mathrm{substrate}}+E_{\mathrm{adsorbate}}\right),$$ (1)

where *E*_substrate+adsorbate_ is the total energy of the substrate with adsorbed molecules. *E*_substrate_ and *E*_adsorbate_ are the energy of the isolated substrate and free molecule, respectively.

### Reaction Free Energy

The computational hydrogen electrode (CHE) model (Norskov et al., 2004) was adopted to evaluate the free energy change during the CO_2_RR process. In the CHE model, the hydrogen atom is in equilibrium with the proton/electron pair at 298.15 K and 1 atm of pressure. In other words, the half chemical potential of gas-phase H_2_ is equal to that of a proton/electron pair at 0 V in an aqueous solution.

The Gibbs free energy change (Δ*G*) for each elementary CO_2_RR step involving proton/electron pair transfer was calculated by the expression (Norskov et al., 2004; Zuluaga and Stolbov, 2011):

$$\Delta G=\Delta E+\Delta ZPE-T\Delta S+\Delta G\text{u}+\Delta {\text{G}}_{\text{pH}},$$ (2)

where Δ*E* is the change of reaction energy based on DFT calculations. Δ*ZPE* and Δ*S* are the change of zero-point energy and entropy, respectively. T refers to the temperature (298.15 K). The zero-point energy (*ZPE*) of adsorbates has been calculated from the vibrational frequencies. For the free molecules (CO_2_, CO, HCOOH, CH_4_, CH_3_OH, *etc.*) the vibrational frequencies and entropies are obtained from the NIST database (http://webbook.nist.gov/chemistry/). Δ*G*_U_ = -n*e*U, where *n* is the number of transferred electrons, *e* is the elementary charge of an electron, and U is the electrode potential vs. RHE. Δ*G*_*pH=*_ 2.303 *k*_B_T *pH*, k*_B_ is the Boltzmann constant. In this work, the value of pH was set as 0 for the acid medium (Faccio et al., 2010; Shang et al., 2010). Approximate solvation corrections with a dielectric constant of *ε* = 80 are applied for the simulation of an aqueous environment (Mathew et al., 2019).

## Result and Discussion

### Adsorption of the Key Intermediates

**FIGURE 1:**
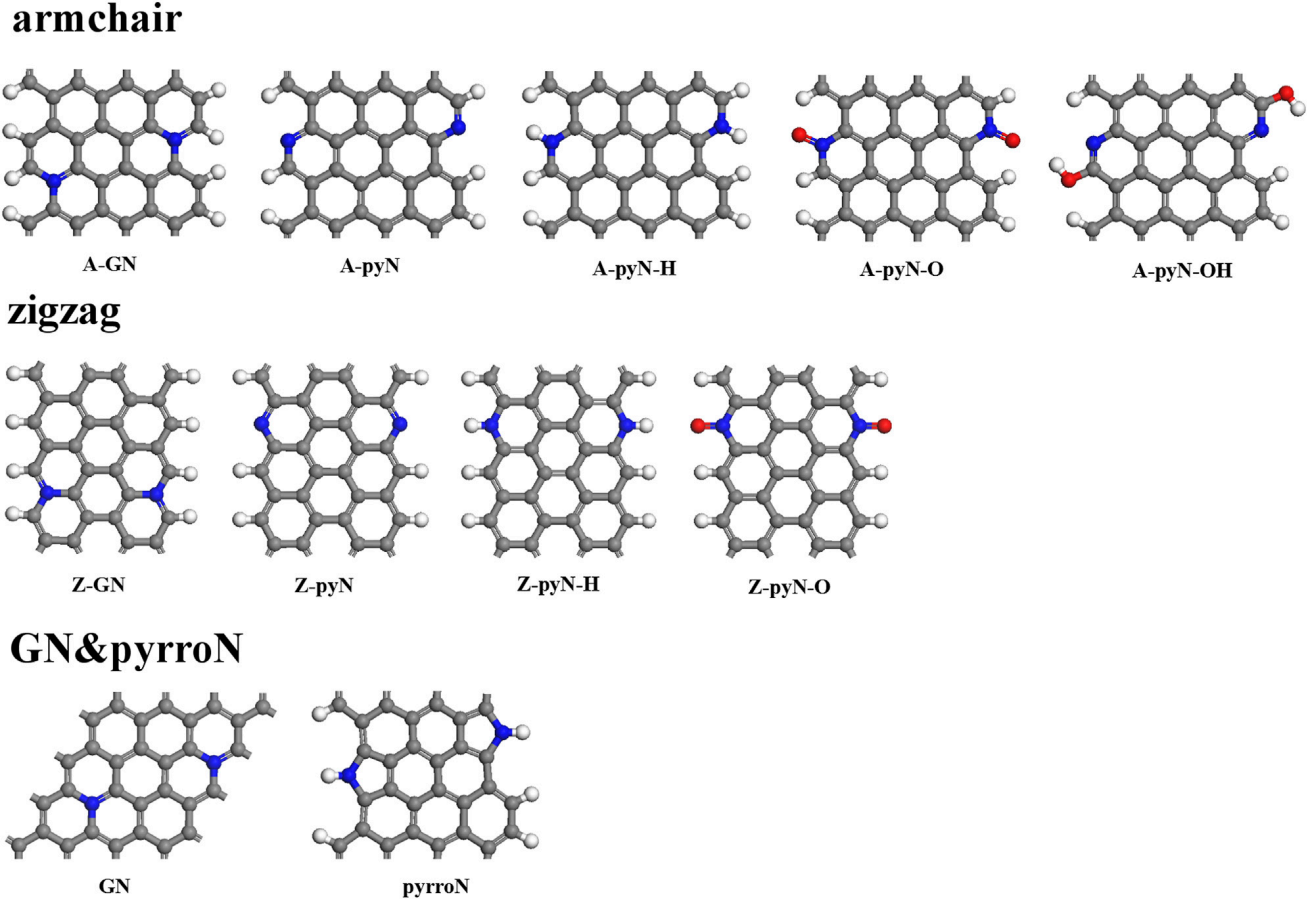
The structures of N-doped graphene. The gray, blue, white, and red balls represent C, N, H, and O atoms, respectively.

In previous reports, the N-doped graphene materials have been widely studied as ORR electrocatalysts, which showed better stability and tolerance to methanol crossover effect than commercial Pt/C catalyst (Geng et al., 2011; Lin et al., 2013; Gong et al., 2009; Qu et al., 2010). Under different temperatures, the synthesizability of each type of the N-doped graphene materials is different. It is relatively easy to synthesize different types of N-doped graphene by controlling the temperature (Lin et al., 2013). The studied structures include five N-doped armchair graphene types, four N-doped zigzag graphene types, in-plane graphitic N (GN), and pyrrolic edge N (PyrroN)-doped graphene. For N-doped armchair graphene, it includes graphitic N (A-GN), pyridinic N (A-pyN), hydrogenated pyridinic N (A-pyN-H), oxidized pyridinic N (A-pyN-O), and pyridinic N hydroxide (A-pyN-OH), as shown in Figure 1. For N-doped zigzag graphene, four structures are considered, i.e., graphitic N (Z-GN), pyridinic N (Z-pyN), hydrogenated pyridinic N (Z-pyN-H), and oxidized pyridinic N (Z-pyN-O). These doped structures could be generated at high temperatures in the pyrolysis process of N-containing compounds (Wu et al., 2011; Li et al., 2012; Wang Q. et al., 2014; Holby et al., 2014).

During the CO_2_RR process on the studied compounds, the intermediates mainly include CO_2_, COOH, HCOO, HCOOH, CO + H_2_O, COHOH, H_2_COO, and COH + H_2_O. By exploring different adsorption sites (N and its adjacent carbon atoms), the most favorable adsorption configurations and sites are obtained (Supplementary Figures 1–8). Since the two main reactions on various N-doped graphene are HCOOH and CO generation pathways, we focus on the adsorption energies of CO_2_, COOH, HCOO, HCOOH, and CO as listed in Table 1, together with the bond distance between the adsorbed intermediates and catalyst surface. It is seen that the adsorption of CO_2_ molecule is weak all the time (-0.06 eV∼ -0.13 eV), and linear structure is maintained above the surface. To achieve high selectivity for HCOOH or CO, COOH (or HCOO) should be adsorbed strongly, but HCOOH or CO should be adsorbed weakly for desorption. Therefore, strong COOH (HCOO) binding but weak HCOOH (CO) adsorption is essential for the formation of HCOOH or (CO) (Sharma et al., 2015; Wu et al., 2015).

**TABLE 1:** The calculated adsorption energies (E_ads_, eV) and the shortest distances (d, Å) between the intermediate and N-doped graphene.

	*CO_2_		*COOH		*HCOO		*HCOOH		*CO	
	E_ads_	D	E_ads_	d	E_ads_	d	E_ads_	d	E_ads_	d
A-GN	−0.13	3.14	−1.26	1.57	__	__	−0.12	2.23	−0.13	3.12
A-pyN	−0.10	3.08	−1.51	1.42	−0.92	1.52	−0.33	1.73	−0.04	3.22
A-pyN-H	−0.10	3.10	−0.20	1.53	__	__	−0.05	1.92	−0.03	3.17
A-pyN-O	−0.11	3.32	−0.95	1.56	−1.29	1.50	−0.40	1.62	−0.08	3.26
A-pyN-OH	−0.11	3.12	−0.96	1.40	−0.84	1.54	−0.24	1.63	−0.07	3.19
Z-GN	−0.10	3.25	−1.99	1.58	−1.87	1.50	−0.16	2.02	−0.10	3.14
Z-pyN	−0.09	3.36	−2.48	1.41	__	__	−0.43	1.64	−0.26	1.37
Z-pyN-H	−0.09	3.19	−0.43	1.58	__	__	−0.13	2.00	−0.09	3.17
Z-pyN-O	−0.08	3.19	−0.59	1.55	__	__	−0.06	2.39	−0.10	3.20
GN	−0.06	3.11	0.34	1.61	__	__	−0.10	2.22	−0.12	3.14
PyrroN	−0.10	3.03	__	__	__	__	__	__	__	__

The “*” denotes the adsorption state of the species.

As shown in Supplementary Figures 2, 3, COOH could not be absorbed on GN and PyrroN, and is weakly adsorbed on A-pyN-H (-0.20 eV), Z-pyN-H (-0.43 eV), and Z-pyN-O (−0.59 eV) (Table 1). For the remaining structures, the adsorption of COOH is relatively strong, with the adsorption energy ranging from −0.95 to −2.48 eV. However, HCOO exists only on four N-doped graphene structures, that is, A-pyN, A-pyN-O, A-pyN-OH, and Z-GN. The adsorption energies for the four structures are in the range of −1.87 eV∼ −0.84 eV (Table 1).

For HCOOH, the adsorption energies for the studied compounds are in the range of −0.43 ∼ −0.06 eV, which are relatively weak and facilitate its desorption from the catalyst surface. Similar to the HCOOH molecule, the adsorption energies of CO are in the range of −0.26 ∼ −0.03 eV (Table 1).

### Reaction Mechanism

**FIGURE 2:**
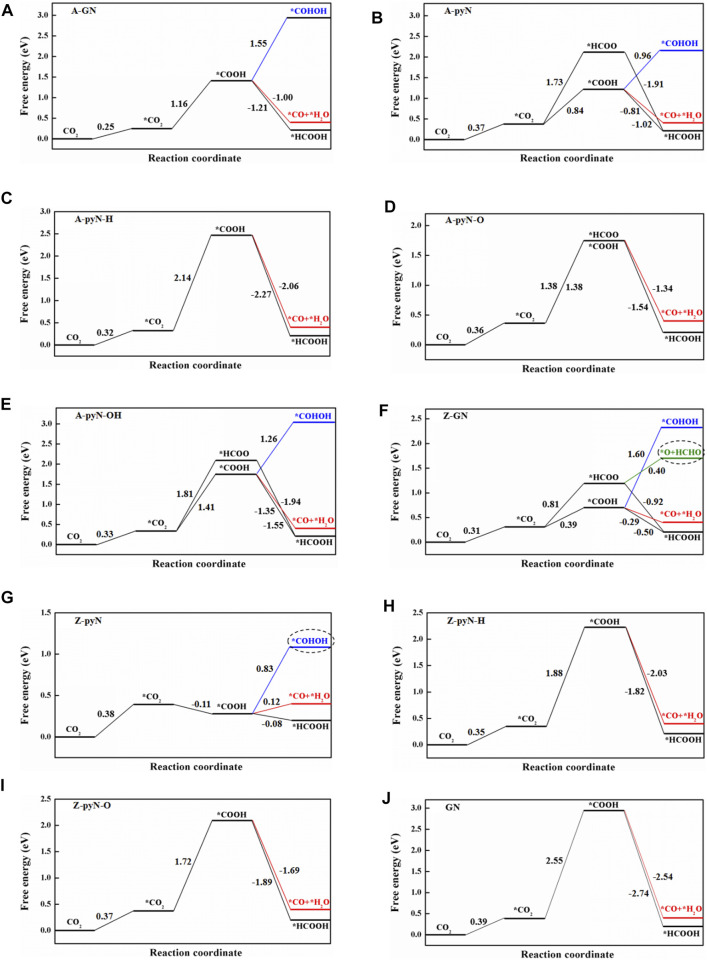
The free energy change for reaction pathways of CO_2_RR on various N-doped graphene. **(A)** A-GN, **(B)** A-pyN, **(C)** A-pyN-H, **(D)** A-pyN-O, **(E)** A-pyN-OH, **(F)** Z-GN, **(G)** Z-pyN, **(H)** Z-pyN-H, **(I)** Z-pyN-O, **(J)** GN.

The possible reaction pathways for the studied compounds are summarized in Figure 2. Based on the computational hydrogen electrode (CHE) model (Norskov et al., 2004), the limiting potential is obtained by U_L_ = −ΔG_MAX/_ *e*, where ΔG_MAX_ denotes the maximum free energy difference between the two successive reaction steps. The reduction step corresponding to the limiting potential is defined as the potential determining step (PDS).

### N-Doped Armchair Graphene Nanoribbons

As shown in Figures 2A–E, the energy of CO_2_ increases by 0.25–0.37 eV from the free molecule to the adsorbed state. After CO_2_ is adsorbed on the catalyst surface, it would be hydrogenated by (H^+^ + e^−^) pair. The formation of an O-H bond would produce COOH, while the formation of the C-H bond would generate the HCOO intermediate.

The reaction of CO_2_+H^+^+e^−^→*COOH on A-GN, A-pyN, A-pyN-H, A-pyN-O, and A-pyN-OH is uphill by 1.16, 0.84, 2.14, 1.38, and 1.41 eV, respectively. For CO_2_+ H^+^+e^−^→*HCOO, the energy increases by 1.73, 1.38, and 1.81 eV for A-pyN, A-pyN-O, and A-pyN-OH, respectively.

The hydrogenation of COOH would generate COHOH, HCOOH, and CO + H_2_O. Due to the large energy increase for producing COHOH, that is, 1.55, 0.96, and 1.26 eV for A-GN, A-pyN, and A-pyN-OH, respectively, further discussion is omitted. In COOH, if the OH moiety binds (H^+^+e^−^), it would produce CO + H_2_O. If the carbon atom in COOH binds (H^+^+e^−^), it would produce HCOOH. The production of HCOOH and CO is all thermodynamically downhill.

Similarly, the hydrogenation of HCOO may produce H_2_COO and HCOOH. As *HCOO→*H_2_COO step is endothermic with a large free energy increase (0.88 eV for A-pyN, 0.93 eV for A-pyN-O, and 1.86 eV for A-pyN-OH), further discussion is not provided. Thus, the final product from HCOO is HCOOH.

As illustrated in Figures 2A–E, the COOH intermediate has better performance in producing HCOOH than HCOO. For CO_2_→ *CO_2_→*COOH→*HCOOH/*CO, the PDS is *CO_2_→*COOH (Table 2), which is in agreement with the previous study (Wu et al., 2015). According to the free energy barrier (Figures 2A–E), A-pyN exhibits the highest catalytic activity toward HCOOH with a free energy barrier of 0.84 eV (Figure 2B). The order of catalytic activity for COOH to HCOOH/CO is A-pyN > A-GN > A-pyN-O > A-pyN-OH > A-pyN-H. In addition, CO_2_→ *CO_2_→*COOH→*CO+*H_2_O is the secondary pathway with slightly larger endothermic energy than CO_2_→ *CO_2_→*COOH→*HCOOH.

**TABLE 2:** Potential determining steps (PDSs), limiting potentials (U_L_/V), and overpotentials (ƞ/V) for CO_2_RR on Z-GN and Z-pyN. U_0_ is the equilibrium potential. Comparison has been made with previous studies. U_L_, U_0_, and ƞ are all vs. the RHE.

	PDS	U_L_	U_0_	ƞ	Product
Z-GN	*CO_2_+H^+^ + e^−^→*COOH	−0.39	−0.25	0.14	HCOOH
Z-GN	*CO_2_+H^+^ + e^−^→*COOH	−0.39	−0.11	0.28	CO
Z-GN	*CO_2_+H^+^ + e^−^→*HCOO	−0.81	−0.07	0.74	HCHO
Z-pyN	CO_2_+H^+^ + e^−^→*CO_2_	−0.38	−0.25	0.13	HCOOH
Z-pyN	CO_2_+H^+^ + e^−^→*CO_2_	−0.38	−0.11	0.27	CO
Z-pyN	*COOH + H^+^ + e^−^→*COHOH	−0.83	0.02	0.81	CH_3_OH
Z-pyN	*COOH + H^+^ + e^−^→*COHOH	−0.83	0.17	0.66	CH_4_
PyrroN3	*COOH + H^+^ + e^−^→HOOH	−0.44	__	__	HCOOH
Edge-2gN	CO_2_+H^+^ + e^−^→*COOH	−0.52	__	__	CO

The “*” denotes the adsorption state of the species.

### N-Doped Zigzag Graphene Nanoribbons

**FIGURE 3:**
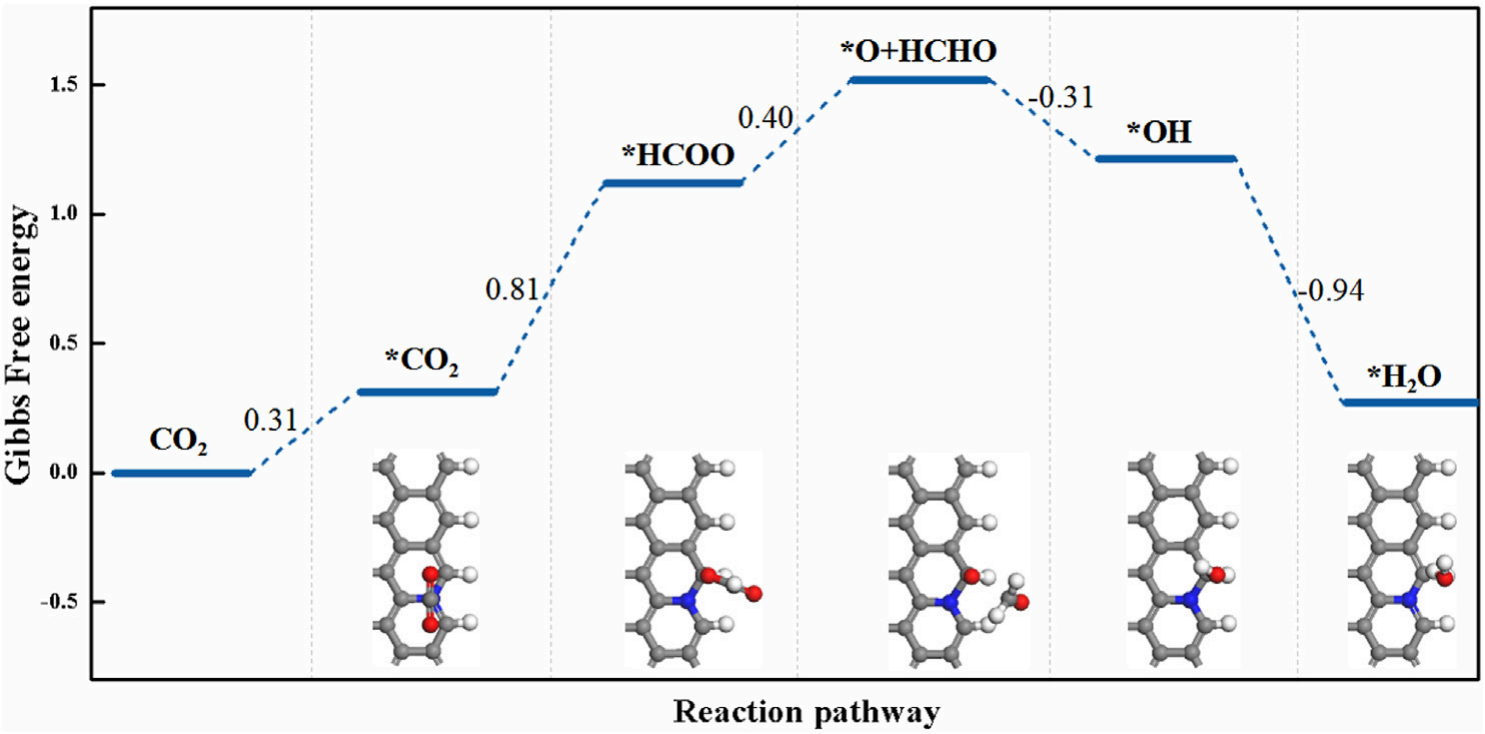
The free energy change for the reaction of CO_2_ + 4H^+^ + 4e^−^→ HCHO + H_2_O on Z-GN.

The reaction pathways on N-doped zigzag graphene nanoribbons (Figures 2F–I) are similar to those on N-doped armchair graphene nanoribbons. The HCOO intermediate could only stably exist on Z-GN among these N-doped zigzag graphene nanoribbons. To produce HCOOH, the CO_2_→*CO_2_→*COOH→*HCOOH pathway is more favorable than the CO_2_→*CO_2_→*HCOO→*HCOOH pathway (Figure 2F). In particular, on Z-GN, the hydrogenation of HCOO generates not only HCOOH but also O + HCHO with an energy barrier of 0.40 eV (Figure 2F). As illustrated in Figure 3, after the formation of O + HCOO, the remaining O atom could be easily hydrogenated into water due to the downhill process. The PDS for producing HCHO is the HCOO formation step with *U*_L_ = -0.81 V.

**FIGURE 4:**
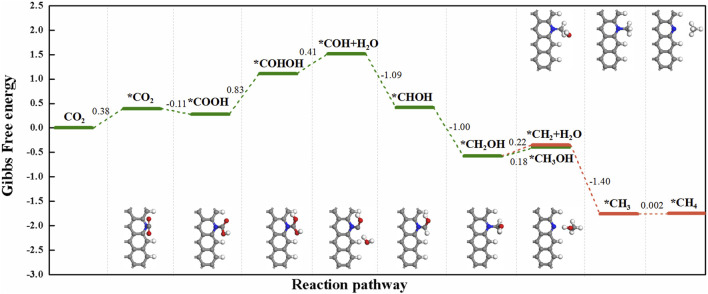
The free energy change for the reaction of CO_2_ + 8H^+^ + 8e^−^→ CH_4_ + H_2_O and CO_2_ + 6H^+^ + 6e^−^→ CH_3_OH + H_2_O on Z-pyN.

For the *CO_2_→*COOH step, it occurred on Z-GN and Z-pyN most easily, in which the energy is uphill by 0.39 eV for Z-GN and downhill by -0.11 eV for Z-pyN, respectively (Figures 2F,G). While for the other two structures, large uphill energy barriers are required, that is, 1.88 eV for Z-pyN-H and 1.72 eV for Z-pyN-O, respectively. After the formation of COOH, its hydrogenation may generate HCOOH, CO + H_2_O, or COHOH, in which the formation of HCOOH is the most favorable, followed by CO + H_2_O and COHOH. Our calculations indicated that the COOH intermediate on Z-pyN needs an energy barrier of 0.83 eV to form COHOH (Figure 4). After the formation of COHOH, an energy increase of 0.41 eV is required to produce COH + H_2_O. The further hydrogenation of COH is relatively easy due to the downhill energy process to release the two competitive final products, that is, CH_3_OH and CH_4_. A previous study indicated that the formation of CH_4_ and CH_3_OH is through CO intermediate (Hori et al., 2008), which is different from our results.

### GN and PyrroN-Doped Graphene

As mentioned above, the pyrrolic N-doped structure has no catalytic activity for CO_2_RR. For GN, the free energy increase is the largest among all the N-doped graphene structures (2.55 eV). Thus, the catalytic activity of GN is omitted.

In a word, for the studied structures, the most favorable product is HCOOH, followed by CO and COHOH. In particular, the formation of HCOOH and CO is competitive since the free energy of CO is more thermodynamically favorable by only about 0.20 eV than that of HCOOH. This energy difference is similar to the value of 0.28 eV reported earlier (Liu et al., 2016). In a word, Z-pyN and Z-GN possess the highest catalytic activity toward HCOOH due to the smallest limiting potential of −0.38 and −0.39 V, respectively (Table 2), which is lower than −0.44 for PyrroN3 (Liu et al., 2016).

### Hydrogen Evolution Reactions

**FIGURE 5:**
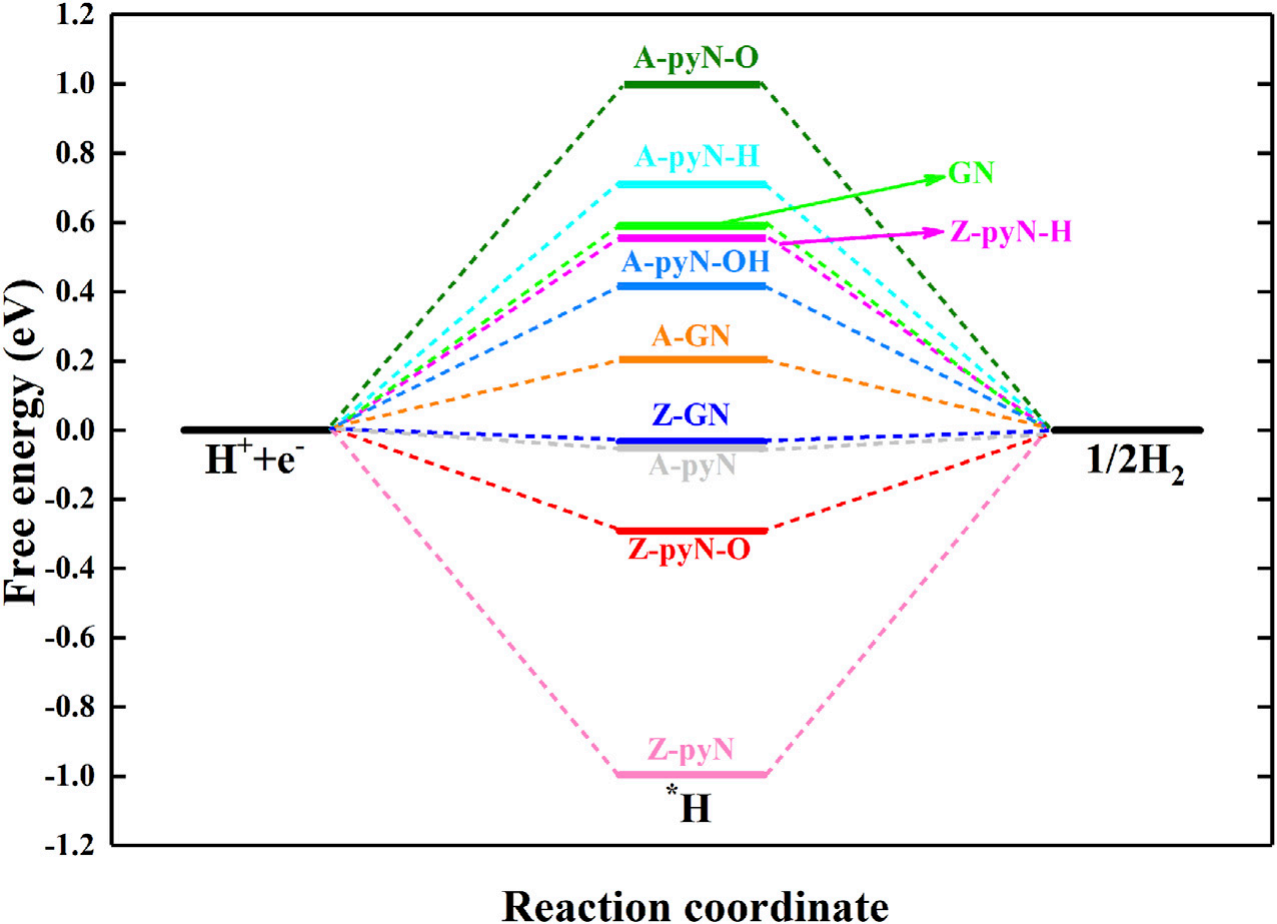
The free energy changes for hydrogen evolution reaction (HER) on various N-doped graphene.

Hydrogen evolution reaction (HER) is the competitive reaction for CO_2_RR since the evolution of H would consume the proton–electron pair (H^+^+e^−^) and passivate the catalytic activity of CO_2_RR. For the studied structures, the results showed that Z-pyN-O and Z-pyN have large energetic downhill for the adsorption of H*, indicating the enhanced HER in thermodynamic (Figure 5). For Z-GN and A-pyN, they have a negligible free energy barrier (0.03 and 0.04 eV) of H*. For the remaining structures, HER is hindered by large free energy barriers. Therefore, for the most favorable Z-pyN and Z-GN, CO_2_RR would be suppressed by HER. However, by choosing a suitable electrolyte, the activation energy of HER would be increased. For instance, according to the expression Δ*G*_*pH=*_ 2.303*k*_B_T pH, in which pH = 0 is selected in the above study, Δ*G*_*pH*_ = 0.42 eV is obtained for pH = 7.0. Thus, the activation energy of HER on Z-GN would be increased from −0.03 to 0.39 eV, comparable to the free energy barrier of 0.38 eV in the CO_2_RR process. Thus, the HER could be suppressed by increasing the pH value for Z-GN. While for Z-pyN, CO_2_RR is passivated by too strong HER. In a word, Z-GN could be selected as a promising nonmetal electrocatalyst for CO_2_RR in generating HCOOH.

## Conclusion

We have performed the DFT method to elucidate the reaction mechanism and activity of CO_2_RR on 11 types of N-doped graphene catalysts. It indicates that for all the studied structures, the formation of HCOOH is the most favorable, followed by CO. Among these structures, Z-pyN- and Z-GN-doped graphene exhibit the best catalytic activity for producing HCOOH with free energy barriers of 0.38 and 0.39 eV, respectively. The potential determining step (PDS) is CO_2_→*CO_2_ for Z-pyN and *CO_2_→*COOH for Z-GN, respectively. Meanwhile, CO is the competitive product which lies 0.20 eV above HCOOH. For the zigzag pyridinic N-doped graphene, it could also produce CH_3_OH and CH_4_ as the minor products which need to overcome an energy barrier of 0.83 eV. The minor product for the zigzag graphitic N-doped graphene is HCHO, with an energy barrier of 0.81 eV. However, for Z-pyN, CO_2_RR is passivated by too strong HER. Meanwhile, by modifying the pH value of electrolyte, Z-GN could be selected as a promising nonmetal electrocatalyst for CO_2_RR in generating HCOOH.

## Data Availability

The original contributions presented in the study are included in the article/Supplementary Material; further inquiries can be directed to the corresponding author.
