# Supplementary material for: Achieving Selective and Efficient Electrocatalytic Activity for CO2 Reduction on N-Doped Graphene
Source: Front Chem. 2021 Aug 19;9:734460. doi: 10.3389/fchem.2021.734460 (PMC8416613; doi:10.3389/fchem.2021.734460)
Supplement: Supplementary file 1 [file DataSheet1.DOCX]

**Achieving selective and efficient electrocatalytic activity for CO_2_ reduction on N doped graphene**

Xiaoxu Sun*

Jiangsu Key Laboratory of New Power Batteries, School of Chemistry and Materials Science, Nanjing Normal University, Nanjing 210023, China.

**Corressponding Author**

xxsun@ciac.ac.cn


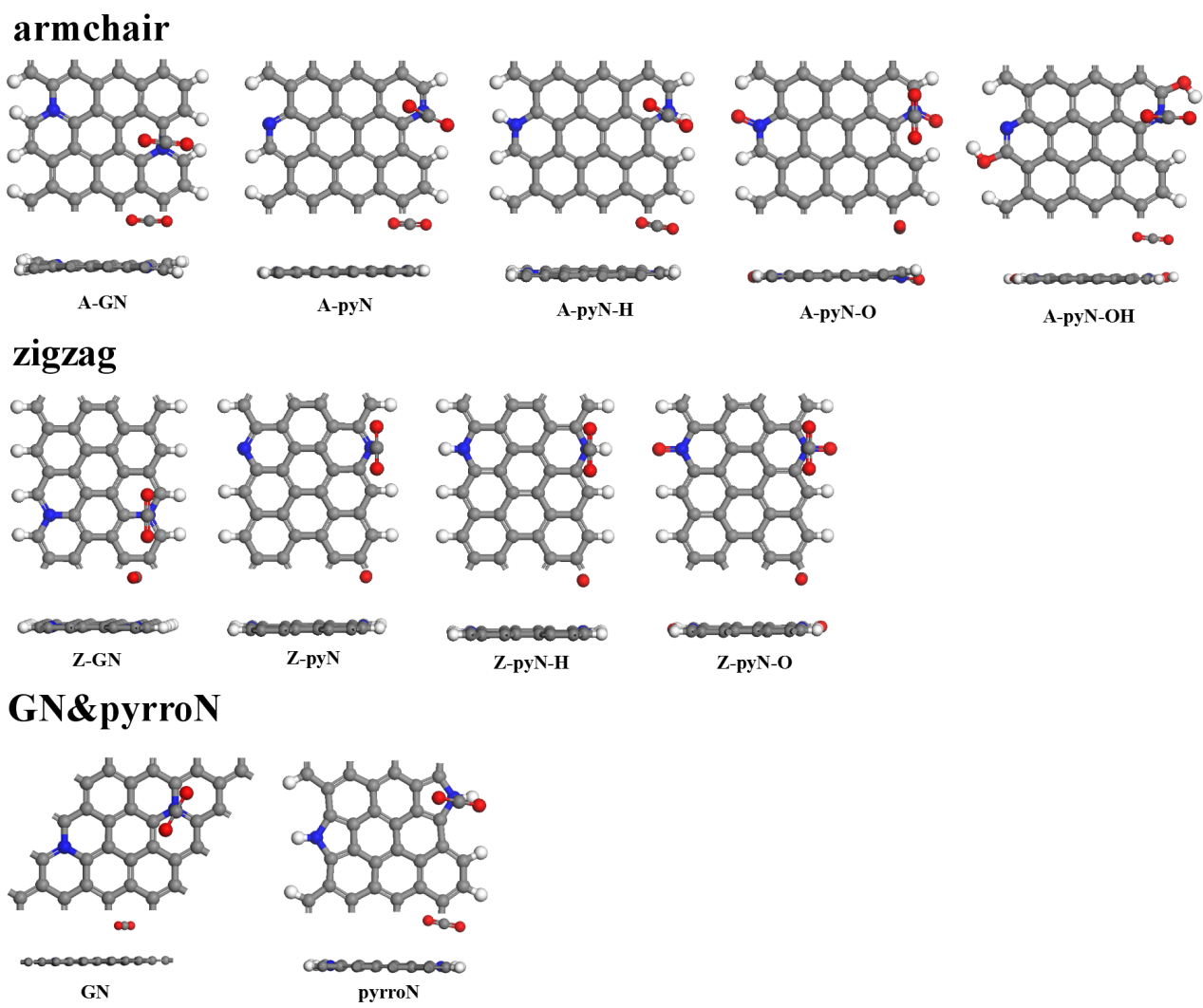


**FIGURE S1** The adsorption structures of CO_2_ on various N doped graphenes.


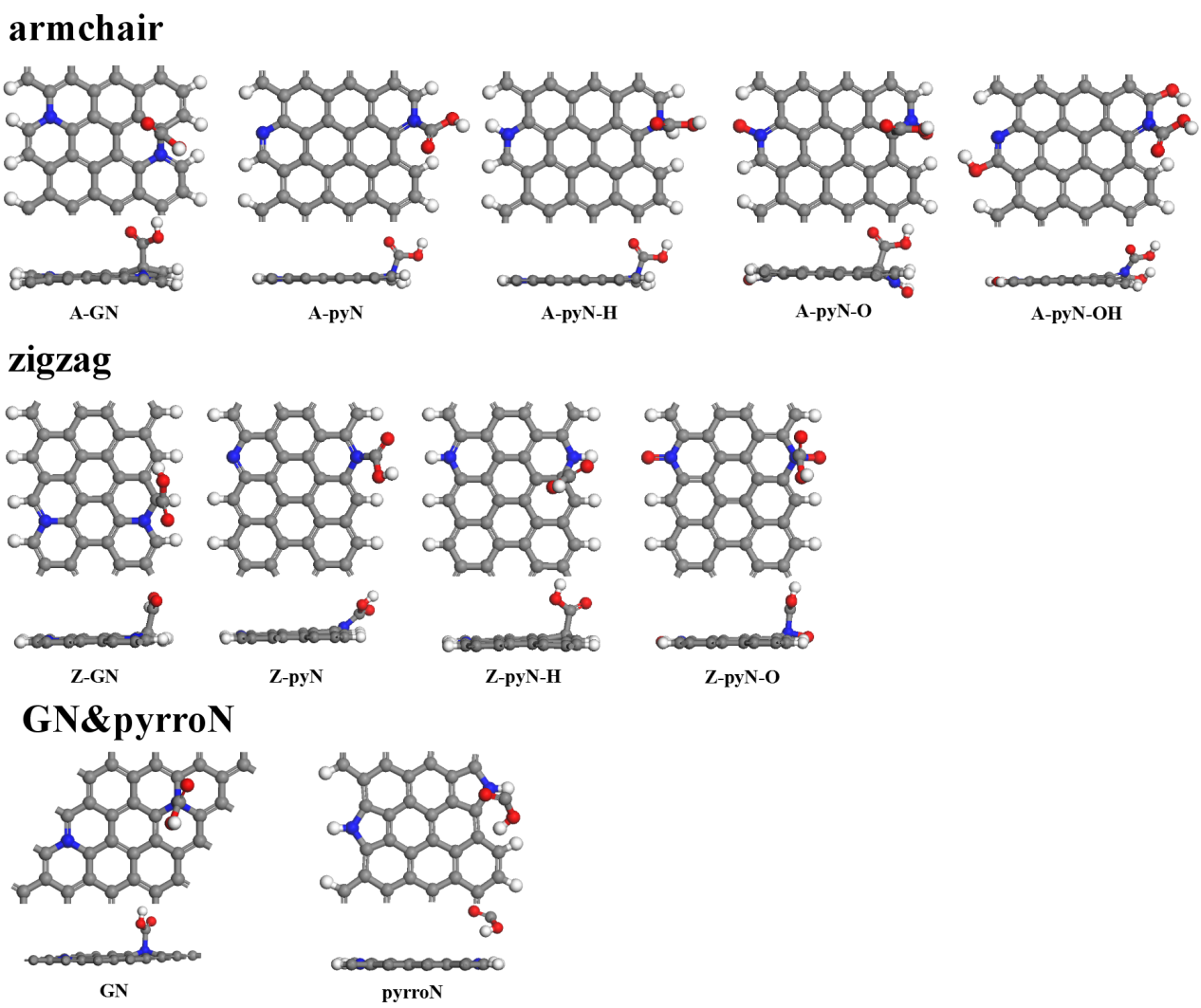


**FIGURE S2** The adsorption structures of COOH on various N doped graphenes.


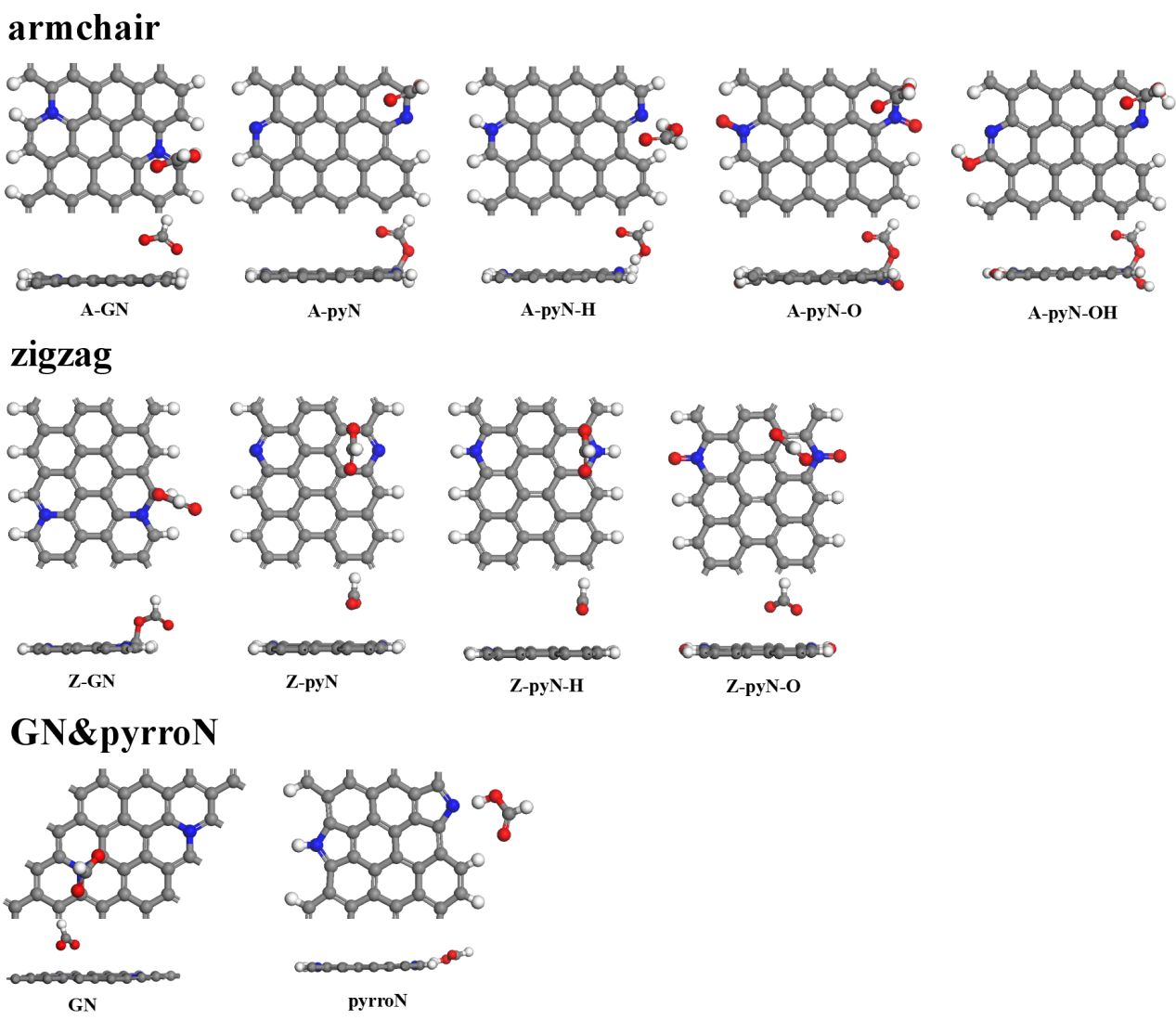


**FIGURE S3** The adsorption structures of HCOO on various N doped graphenes.


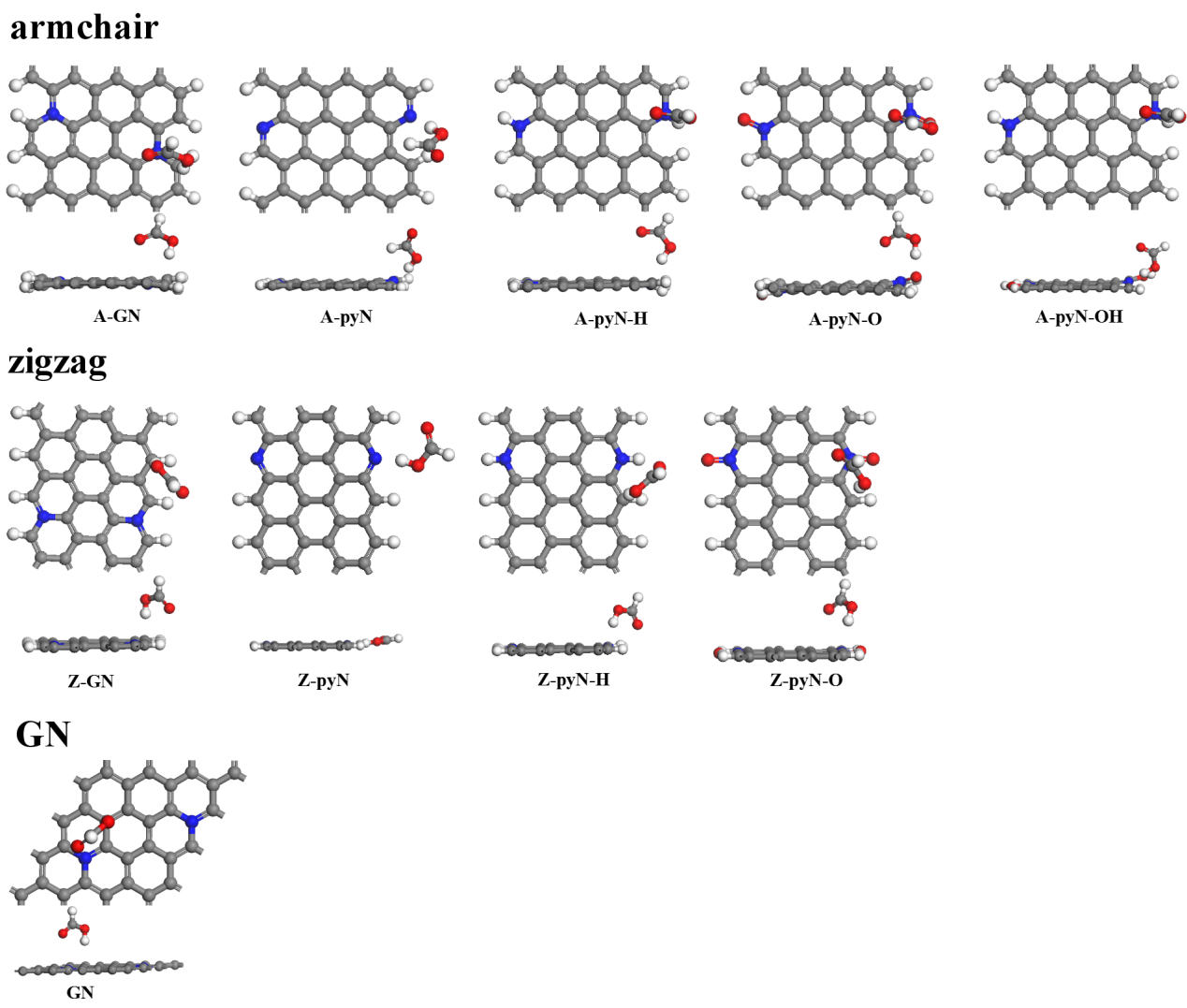


**FIGURE S4** The adsorption structures of HCOOH on various N doped graphenes.


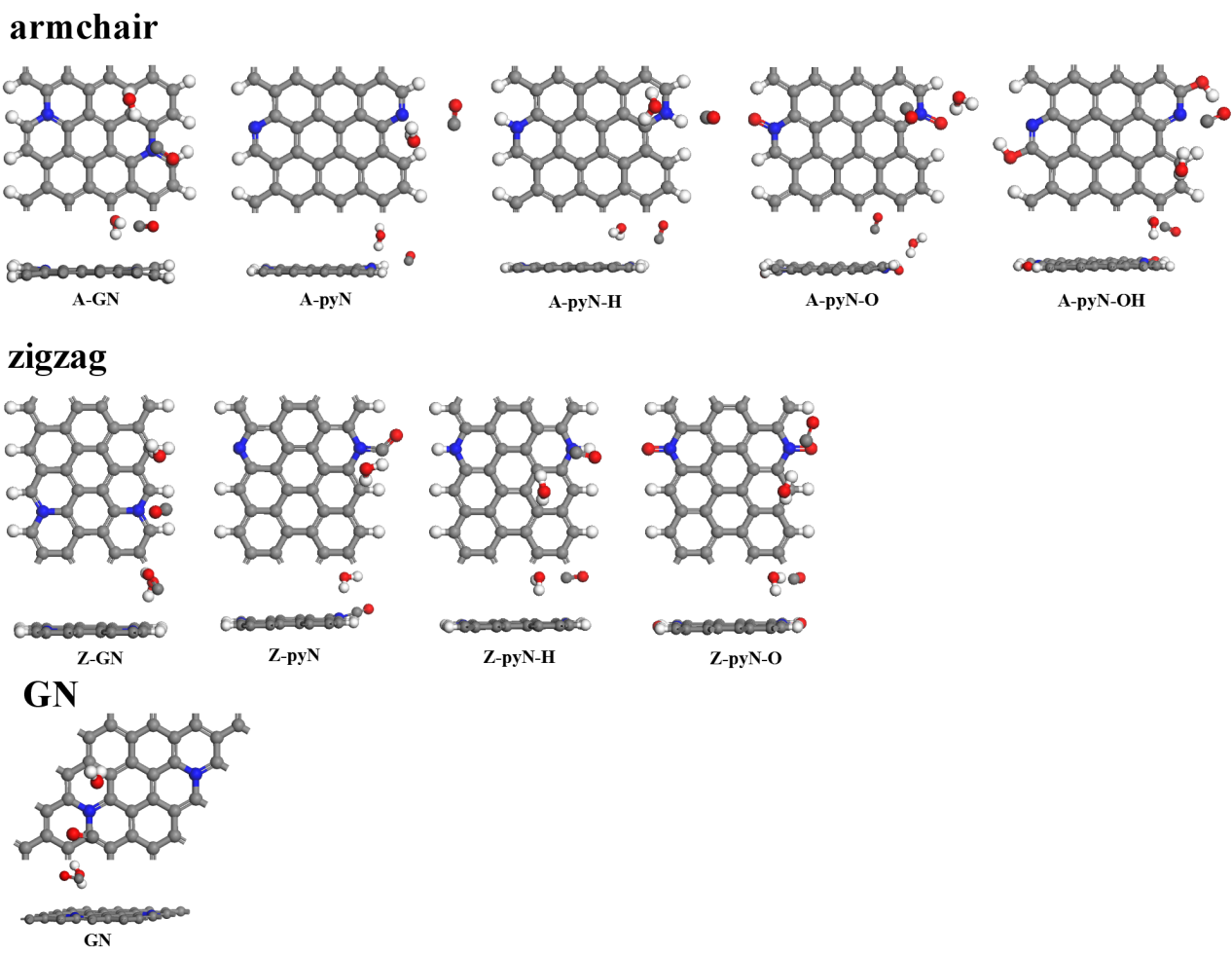


**FIGURE S5** The adsorption structures of CO+H_2_O on various N doped graphenes.


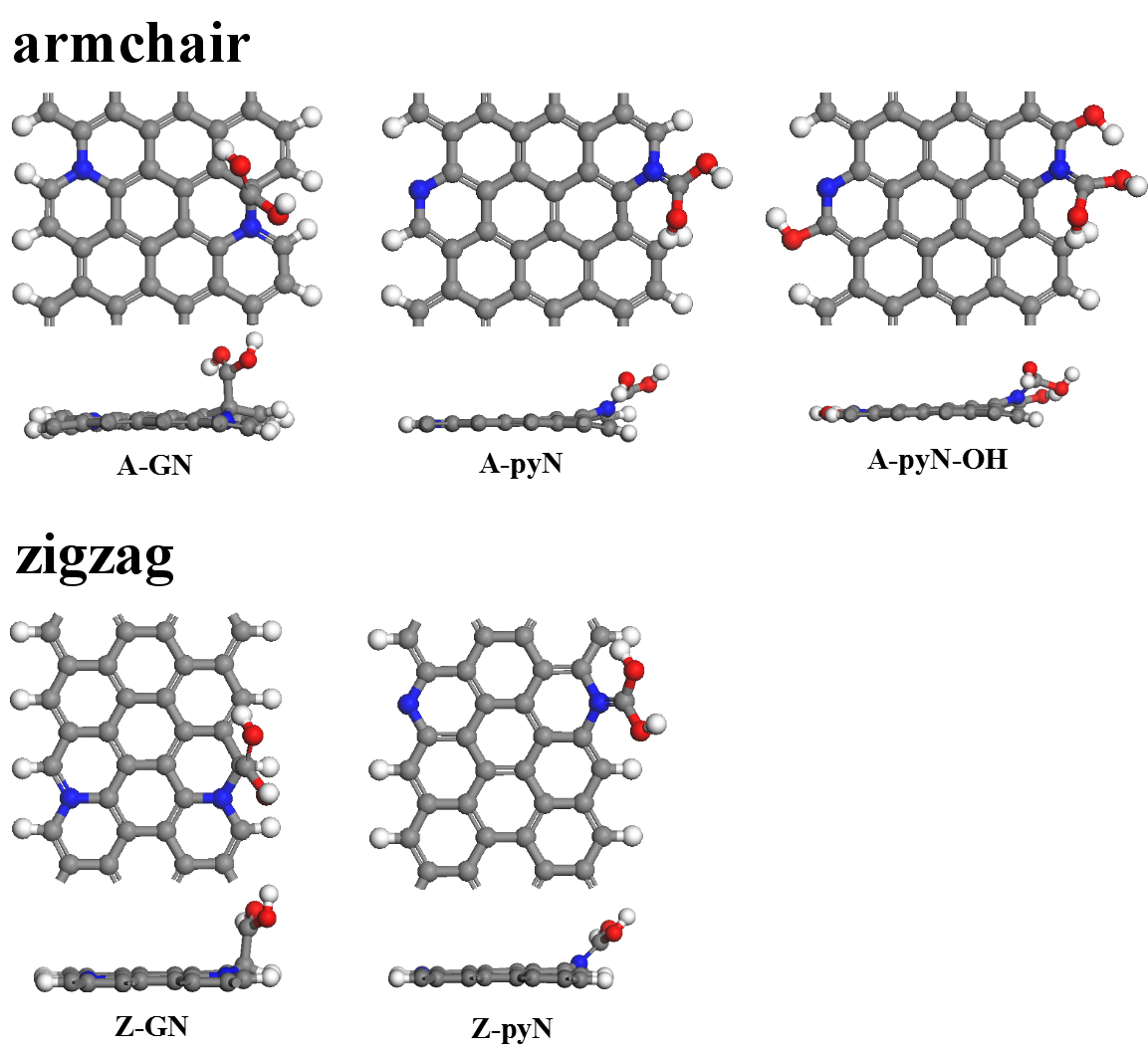


**FIGURE S6** The adsorption structures of COHOH on various N doped graphenes.


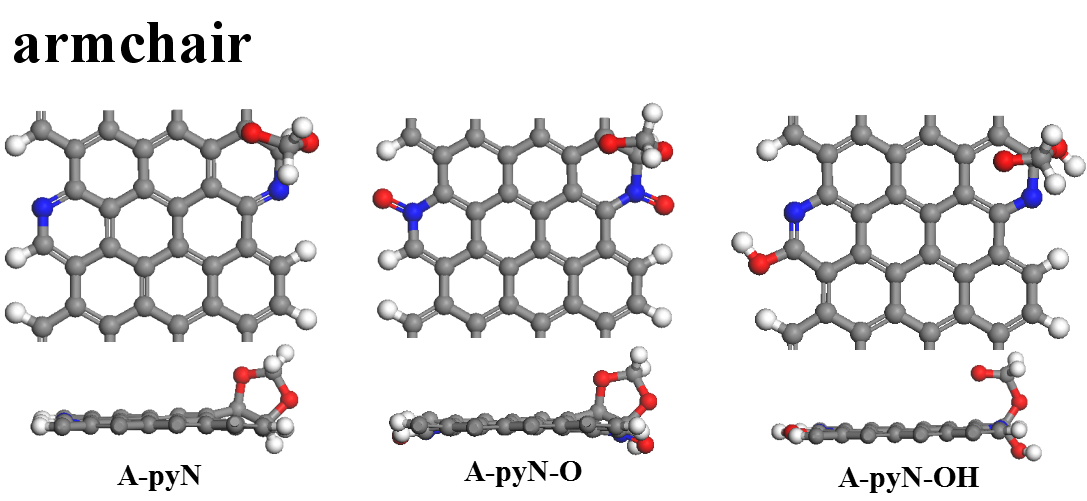


**FIGURE S7** The adsorption structures of H_2_COO on various N doped graphenes.


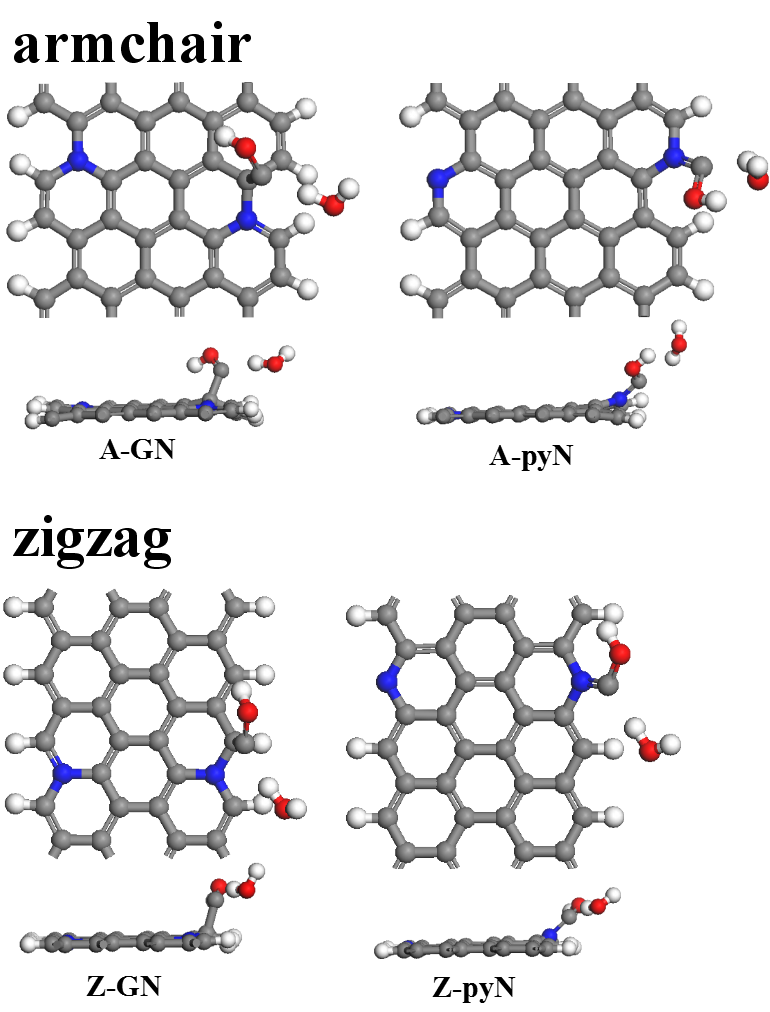


**FIGURE S8** The adsorption structures of COH+H_2_O on various N doped graphenes.
